# Effects of Leucine Supplementation in Older Adults with Sarcopenia: A Meta-Analysis

**DOI:** 10.3390/nu17152413

**Published:** 2025-07-24

**Authors:** Chienhsiu Huang, Min-Hong Hsieh

**Affiliations:** 1 Department of Internal Medicine, Dalin Tzu Chi Hospital, Buddhist Tzu Chi Medical Foundation, Dalin Town 622401, Taiwan; hgssport@yahoo.com.tw; 2 Department of Orthopedics, Dalin Tzu Chi Hospital, Buddhist Tzu Chi Medical Foundation, Dalin Town 622401, Taiwan; 3 School of Medicine, Tzu Chi University, Hualien 97004, Taiwan

**Keywords:** sarcopenia, leucine, older adult, muscular strength, muscle mass, physical performance

## Abstract

**Background and Objectives:** Research on the impact of leucine on older sarcopenic patients is scarce, and investigations on this subject have led to contradictory findings in the literature. Our goal was to compile data from the available studies in the literature to explore the effect of leucine supplementation on parameters associated with sarcopenia in elderly individuals. **Methods:** The meta-analysis included older persons over 65 years of age who were recruited on the basis of the European Working Group on Sarcopenia in Older People sarcopenia criteria. Studies that were included were those in which at least one sarcopenia criterion was measured, including grip strength, appendicular skeletal muscle mass/height^2^, gait speed, and the short physical performance battery index. **Results:** The meta-analysis included ten randomized controlled trials and one prospective study. The leucine group included 566 participants, whereas the placebo group included 567 patients. Patients receiving leucine and patients receiving a placebo had significantly different handgrip (*p* = 0.03), appendicular skeletal muscle mass/height^2^ (*p* = 0.0.2), and gait speed (*p* = 0.008). Patients received a high dosage of leucine, and there was a significant difference in the appendicular skeletal muscle mass/height^2^ (*p* = 0.02) and gait speed (*p* = 0.01) between the high dosage of the leucine group and the control group. When vitamin D was combined with leucine, the appendicular skeletal muscle mass/height^2^ (*p* = 0.03) significantly differed between the leucine group receiving vitamin D and the control group. **Conclusions:** Low-quality evidence was found that older sarcopenic patients receiving leucine may show trends toward improved skeletal muscle strength, skeletal muscle quality, and physical performance. The capacity of leucine supplementation to have a beneficial therapeutic impact in older sarcopenic individuals is restricted when it is used alone without concurrent additional therapy.

## 1. Introduction

### 1.1. Definition of Sarcopenia

The European Working Group on Sarcopenia in Older People (EWGSOP) published consensus diagnostic criteria and a useful clinical definition for sarcopenia in older adults which were developed between 2009 and 2010. Sarcopenia is a syndrome characterized by the progressive and generalized loss of skeletal muscle mass (SMM) and strength according to the EWGSOP. The EWGSOP suggests that low muscle mass and low muscle function be used as diagnostic criteria for sarcopenia. When all three conditions—low muscle mass, low muscle strength, and low physical performance—are present, severe sarcopenia may be diagnosed [1]. The International Working Group on Sarcopenia (IWGS) adopted a similar strategy in 2009 and provided a consensus definition of sarcopenia as the age-related loss of SMM and skeletal muscle function, suggesting that a diagnosis of sarcopenia be made in individuals with a low whole-body or appendicular fat-free mass along with impaired physical functioning [2].

The EWGSOP updated the definition of sarcopenia in 2019. Sarcopenia is a generalized skeletal muscle disease that progresses over time, and low muscle strength is the main indicator of sarcopenia according to the EWGSOP2. Muscle strength is known to predict adverse outcomes more accurately than muscle mass is [3,4,5,6,7].

### 1.2. Current Management of Sarcopenic Patients

The EWGSOP recommends that healthcare professionals increase awareness of sarcopenia and encourage early diagnosis and treatment for sarcopenic patients [3]. Sarcopenia is linked to significant adverse consequences, such as fractures, falls, higher hospitalization rates, longer hospital stays, a greater chance of needing care, and earlier mortality [8,9,10,11]. Sarcopenia is a prevalent condition that affects 5–10% of people over 65 years of age, with rates exceeding 30% in hospitals or care facilities [12,13]. There are currently no effective pharmaceutical treatments for sarcopenia [14]. The best way to prevent and treat sarcopenia is to consume enough protein in the diet and engage in resistance training. Resistance is the most common evidence-based intervention for preventing or treating sarcopenia. However, not every elderly sarcopenic patient is able or ready to engage in resistance training. Given the prevalence of sarcopenia among the elderly population, access to resistance training is inadequate to meet the demand [15,16,17].

Previous studies have demonstrated that maintaining SMM in older individuals requires the consumption of an adequate amount of total protein in the diet [18]. For many older persons who are unable to exercise, nutritional interventions are still the most promising therapeutic and preventative approaches [19,20]. Increasing protein intake is a good way to prevent sarcopenia to increase muscle mass and muscle strength [21,22]. In addition to nutritional interventions, supplements are useful and important in the context of sarcopenia. Recent evidence highlights the role of supplements, including leucine and other bioactive compounds, in supporting muscle health [23].

### 1.3. Effects of Leucine Administration in Sarcopenic Patients

Leucine has been demonstrated to modify skeletal muscle protein turnover by increasing protein synthesis and reducing proteolysis [24,25,26]. Additionally, leucine can promote the release of insulin from pancreatic cells, a crucial anabolic signal in skeletal muscle, and enhance the absorption of glucose by skeletal muscle [27]. The mammalian target of the rapamycin pathway is one way in which leucine regulates muscle function [28]. Dietary leucine supplementation has been studied as a strategy to increase the production of muscle proteins [29]. Therefore, one of the most common treatments for sarcopenia in elderly individuals is supplementation with leucine- or leucine-enriched protein [30,31,32,33]. Martínez-Arnau et al. (2020) [31] included individuals aged 65 years or older in their study and randomly assigned them to a 13-week parallel group intervention consisting of 6 g of leucine daily or 6 g of lactose daily. Leucine administration considerably enhanced walking time. There was no significant difference in the SMM index or handgrip strength between the two groups [31]. The LACE trial (2022) [34] showed that leucine did not increase the short physical performance battery (SPPB) score in individuals randomly assigned to receive 2.5 g of oral leucine three times daily as opposed to placebo. Additionally, there was no discernible treatment advantage in terms of muscle mass, grip strength, gait speed, or chair stand time [34]. Eighty-one sarcopenic participants aged 65 years or older were enrolled in the study by Mori et al. (2022) [35]. The research findings indicated a significant increase in both handgrip strength and appendicular skeletal muscle mass index (ASMI) values in the groups that received resistance training and leucine-enriched whey protein supplementation compared with the baseline values. At 24 weeks of the detraining phase, the mean SMM index and mean handgrip strength were substantially greater in the groups that received leucine-enriched whey protein supplementation and resistance training than in the group that received resistance training. The author concluded that older persons with sarcopenia may benefit from the long-term maintenance of resistance exercise and leucine-enriched whey protein supplementation [35]. Park et al. reported that leucine dose and grip strength were positively correlated. Grip strength increased by 0.796 kg for every 1 g/day increase in the leucine dosage [36]. Vitamin D has been demonstrated to be effective for muscle recovery in various ways [37]. Its synergistic effect with leucine in promoting protein anabolism is well documented [38]. In older adults, vitamin D may also enhance muscular function and increase physical activity [39].

### 1.4. Aim of This Meta-Analysis

However, research on the impact of leucine on older sarcopenic patients is scarce, and investigations on this subject have led to contradictory findings in the literature. The effect of leucine supplementation is controversial precisely because prior meta-analyses have yielded contradictory results. We conducted a meta-analysis to determine whether leucine-rich protein supplements can increase muscular strength, muscle quantity, and physical performance in older people with sarcopenia. Our goal was to compile data from the available studies in the literature to explore the effect of leucine supplementation on parameters associated with sarcopenia in elderly individuals.

## 2. Methods

### 2.1. Search Strategy

The PRISMA-P criteria were followed in this meta-analysis. From 1 January 2000 to 31 July 2024, the following search terms were used to search the PubMed, Web of Science, and Cochrane Library databases: (sarcopenia OR frailty) AND (leucine OR leucine-enriched protein OR whey protein) AND (muscle mass OR muscle quantity OR muscle strength OR muscle function OR physical performance).

### 2.2. Study Selection and Data Extraction

To determine the eligibility of the identified trial reports, each study was independently screened and reviewed by two authors. Articles containing relevant terms in each database were identified and imported into Endnote Library for the deletion of duplicate records. After excluding duplicates, the two authors screened the titles and abstracts of all the studies retrieved to identify eligible records. After excluding irrelevant studies, all of the relevant articles were reviewed by reading the full texts to determine eligibility. Data regarding the author, year of publication, country, total number of patients receiving leucine supplementation, total number of patients receiving placebo, daily leucine supplement dose (grams), duration of the intervention, whether exercise training was received, and whether vitamin D was received were extracted from the eligible full-text articles.

Differences in the means and standard deviations (SDs) from baseline to the end of the study were used to compare the leucine supplementation and control groups. The final mean minus the baseline mean was used to determine the mean change values. The following formula was used to convert 95% confidence intervals to SDs where there was no description of the SDs in the included articles: 95% confidence interval = mean change value ± 1.96 [SD/(number of patients)^1/2^]. SD change values were estimated from the baseline and final SDs via the following formula, which was derived from the Cochrane Handbook for Systematic Review of Interventions [40,41]:

SD change values = [(SD_baseline_)^2^ + (SD_final_)^2^ − 2 × correlation × SD_baseline_ × SD_final_]^1/2^

We used 0.8 as the assumed correlation.

### 2.3. Inclusion and Exclusion Criteria

This study included older persons over 65 years of age who were recruited on the basis of the EWGSOP2 sarcopenia criteria. We used the following inclusion criteria for every publication that was examined to address our research questions: (1) randomized controlled trials (RCTs), prospective studies, and retrospective studies that compared leucine with a control intervention in older sarcopenic individuals aged 65 years or older; (2) studies published in English; (3) studies in which at least one sarcopenia criterion was measured, including grip strength (kg), appendicular skeletal muscle mass (ASMM)/height^2^ (kg/m^2^), gait speed (meter/second), and the short physical performance battery (SPPB) index (point score); and (4) studies that were not case reports, conference abstracts, letters to the editor, reviews, comments, basic scientific publications, or protocols. Only the most recent update was included when studies involving the same patient group were identified. Studies were excluded if they focused on one group of patients with chronic comorbidities and obesity.

### 2.4. Quality Assessment and Statistical Analysis

We assessed the risk of bias in each study using the Cochrane Risk-of-Bias Tool 2.0 for RCTs. RevMan 5.4 and Cochrane Review Manager software were used for the statistical analyses. Fixed effects and random effects were utilized for data analysis. Statistical heterogeneity was evaluated using the *Q* test and *I*^2^ statistic. We tabulated the study intervention features and compared them to the scheduled groups for each synthesis using forest plots. The funnel plot was examined to determine the degree of publication bias. Quality of the evidence was ranked based on the risk of bias according to the Grading of Recommendations Assessment, Development and Evaluation (GRADE) approach at the outcome level [42,43].

## 3. Results

**Figure 1:**
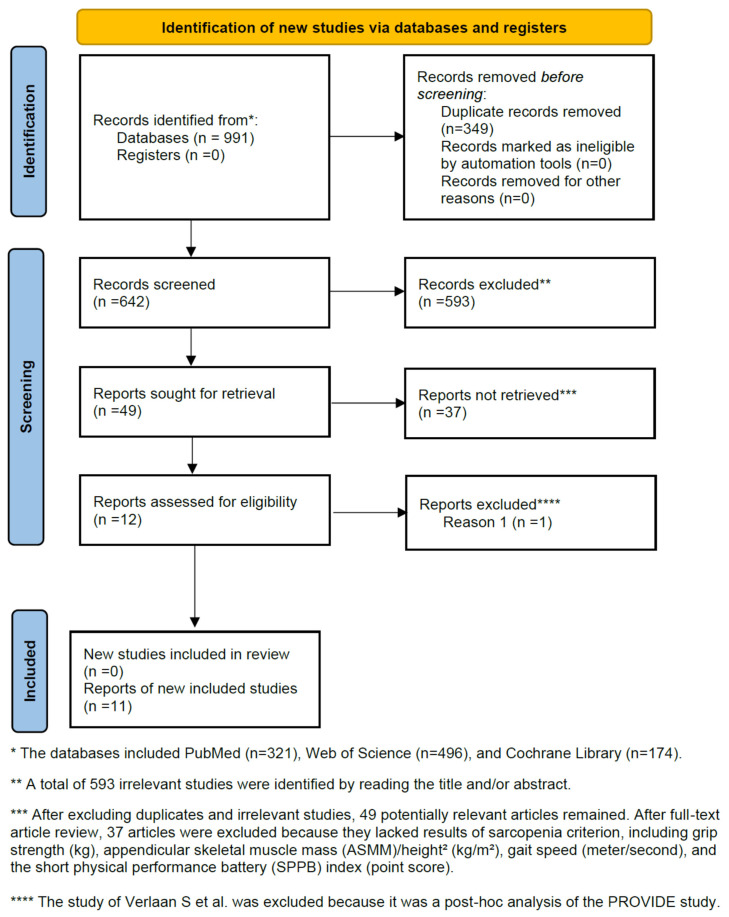
Flow diagram of the study selection process.

The details of the study selection process are shown in Figure 1. After excluding duplicates and irrelevant studies, 49 potentially relevant articles remained. After a full-text article review, 37 studies were excluded because they lacked results comparing the outcomes of the leucine administration group with those of the control group in older sarcopenic adults. The study by Verlaan S et al. was excluded because it was a post hoc analysis of the PROVIDE study [44]. Ultimately, this meta-analysis included ten RCTs [31,32,33,34,35,45,46,47,48,49] and one prospective study [50].

**Figure 2:**
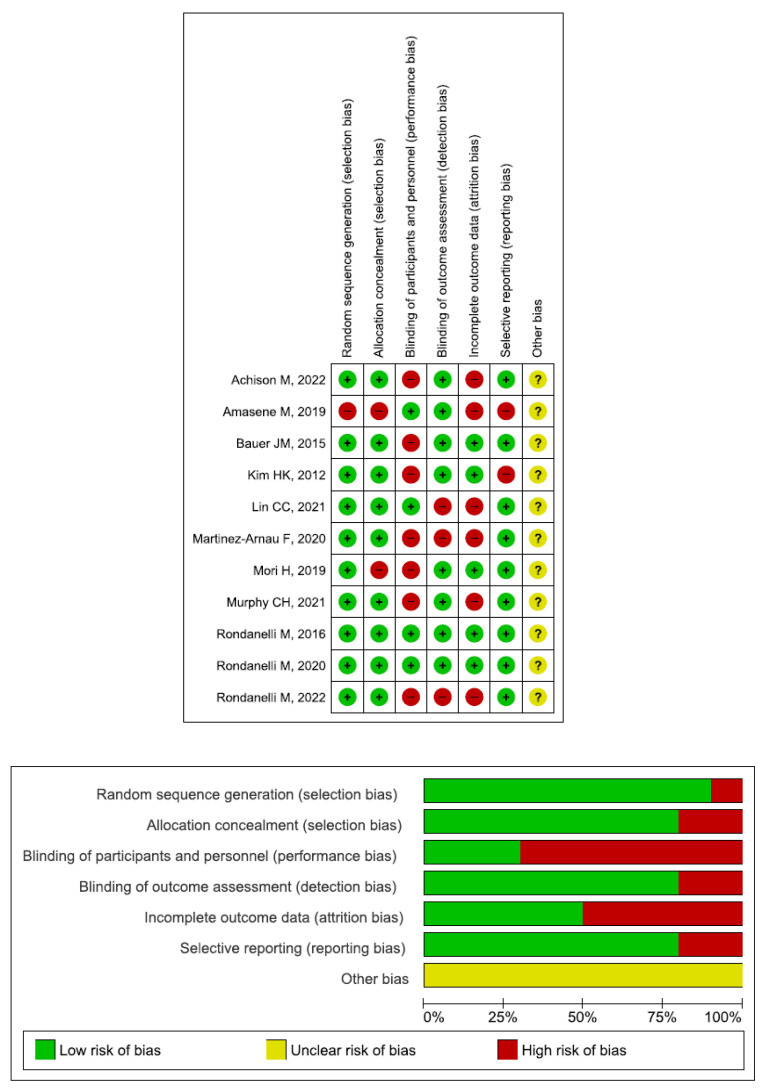
The risk of bias assessment of the ten randomized controlled trials [31,32,33,34,35,45,46,47,48,49,50].

The leucine group included 566 participants, whereas the placebo group included 567 patients. Table 1 displays the main characteristics of the eleven included studies. Figure 2 displays the risk of bias assessment of the ten RCTs. The prospective studies had a high risk of bias.

**Figure 3:**
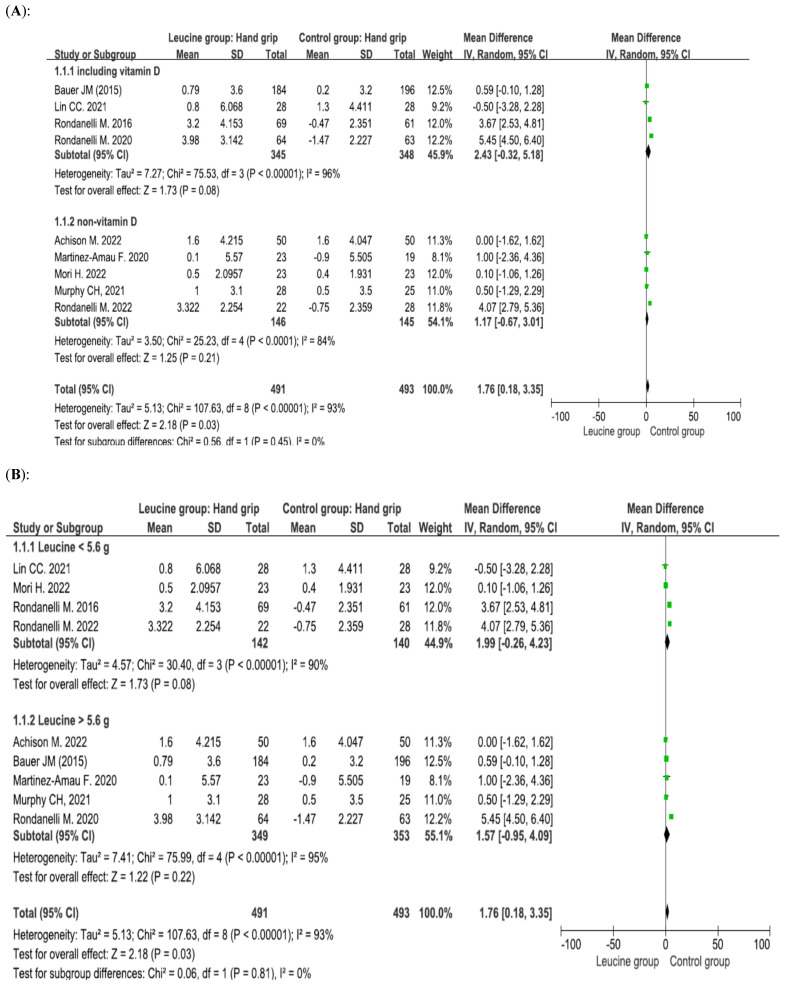
Forest plots assessing the effect of leucine supplementation on handgrip strength in patients with sarcopenia by (**A**) vitamin D supplementation status and (**B**) leucine dose [31,32,34,35,46,47,48,49,50].

Nine studies involving 984 patients (491 patients receiving leucine and 493 control patients) reported handgrip strength, and the difference between the groups was significant (*p* = 0.03, mean difference = 1.76) [31,32,34,35,46,47,48,49,50]. Subgroup analysis of patients who concurrently received vitamin D was performed. When vitamin D was combined with leucine, there was no significant difference between the leucine group and the control group (*p* = 0.08, mean difference = 2.43). When vitamin D was not combined with leucine, there was no significant difference between the leucine group and the control group (*p* = 0.21, mean difference = 1.17) (Figure 3A). On the basis of the daily amount of leucine supplementation, we separated the studies into two groups for analysis: low-dose (less than 5.6 g) and high-dose (greater than 5.5 g) groups. Subgroup analysis of the daily amount of leucine supplementation was performed. When patients received a low dosage of leucine, there was no significant difference between the leucine group and the control group (*p* = 0.08, mean difference = 1.99). When patients received a high dosage of leucine, there was no significant difference between the leucine group and the control group (*p* = 0.22, mean difference = 1.57) (Figure 3B). Leucine dosage did not affect handgrip strength in older sarcopenic adults. Leucine combined with vitamin D did not affect handgrip strength in older sarcopenic adults either.

**Figure 4:**
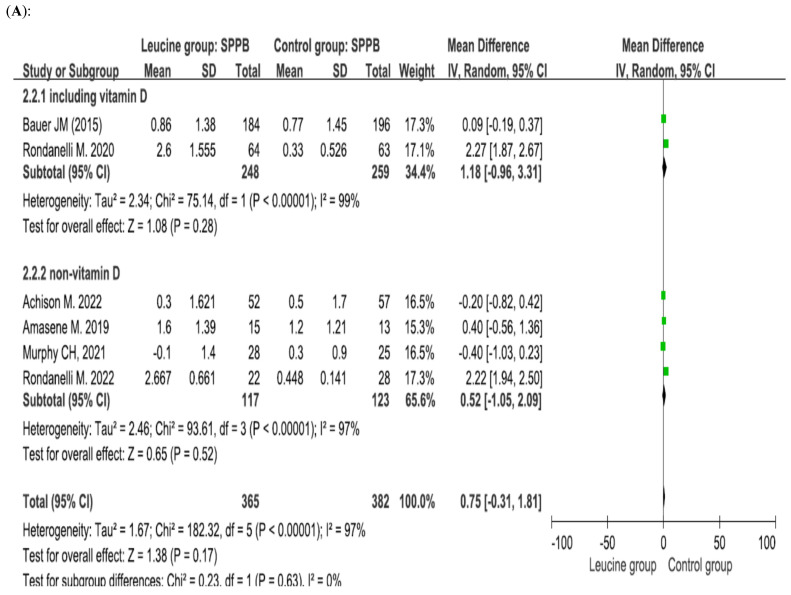

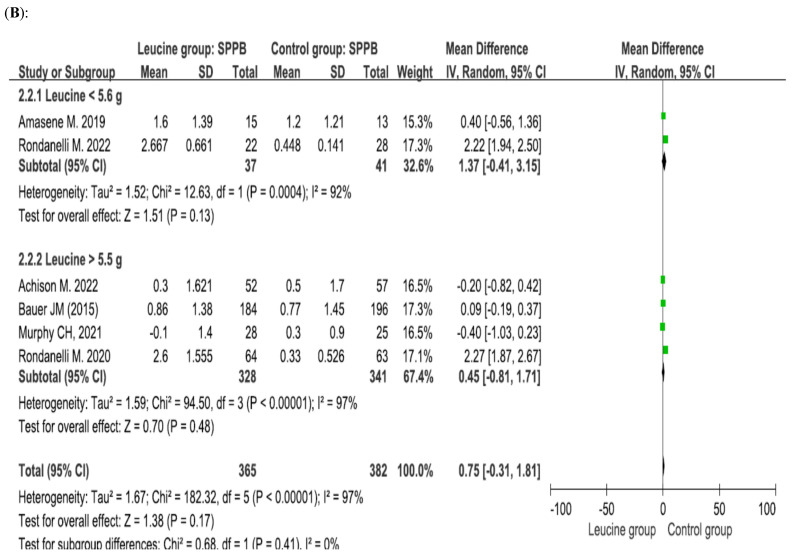
Forest plots assessing the effect of leucine supplementation on SPPB scores in patients with sarcopenia by (**A**) vitamin D supplementation status and (**B**) leucine dose [30,32,33,34,46,48,49].

Six studies involving 747 patients (365 patients receiving leucine and 382 control patients) reported SPPB scores, and the difference between the groups was not significant (*p* = 0.17, mean difference = 0.75) [32,33,34,46,47,49]. Subgroup analysis of patients who concurrently received vitamin D was performed. When vitamin D was combined with leucine, there was no significant difference between the leucine group and the control group (*p* = 0.28, mean difference = 1.18]. When vitamin D supplementation was not combined with leucine, there was no significant difference between the leucine group and the control group (*p* = 0.52, mean difference = 0.52) (Figure 4A). Subgroup analysis of the daily amount of leucine was performed. When patients received a low dose of leucine, there was no significant difference between the leucine group and the control group (*p* = 0.13, mean difference = 1.37). When patients received a high dose of leucine, there was no significant difference between the leucine group and the control group (*p* = 0.48, mean difference = 0.45) (Figure 4B).

**Figure 5:**
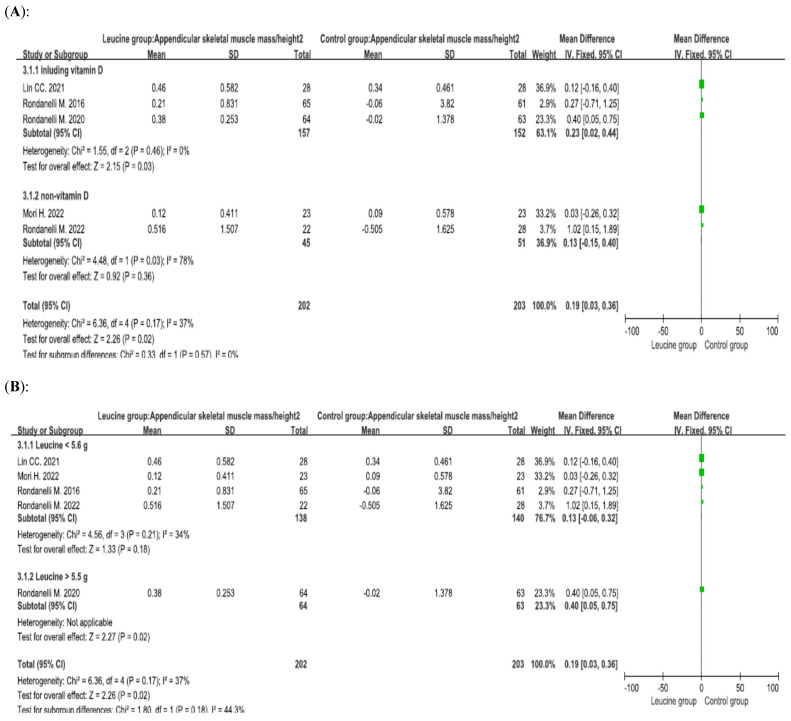
Forest plots assessing the effect of leucine supplementation on appendicular skeletal muscle mass/height^2^ in patients with sarcopenia by (**A**) vitamin D supplementation status and (**B**) leucine dose [35,47,48,49,50].

Five studies involving 405 patients (202 patients receiving leucine and 203 control patients) reported the ASMM/height^2^, and the difference between the groups was significant (*p* = 0.02, mean difference = 0.19) [35,47,48,49,50]. Subgroup analysis of patients who concurrently received vitamin D was performed. Compared with the control group, the leucine supplementation group presented significant improvements when vitamin D was combined with leucine (*p* = 0.03, mean difference = 0.23). When vitamin D was not combined with leucine, there was no significant difference between the leucine group and the control group (*p* = 0.36, mean difference = 0.13) (Figure 5A). Subgroup analysis of the daily amount of leucine was performed. When patients received a low dose of leucine, there was no significant difference between the leucine group and the control group (*p* = 0.18, mean difference = 0.13). When patients received a high dose of leucine, there was a significant difference between the leucine group and the control group (*p* = 0.02, mean difference = 0.40) (Figure 5B).

**Figure 6:**
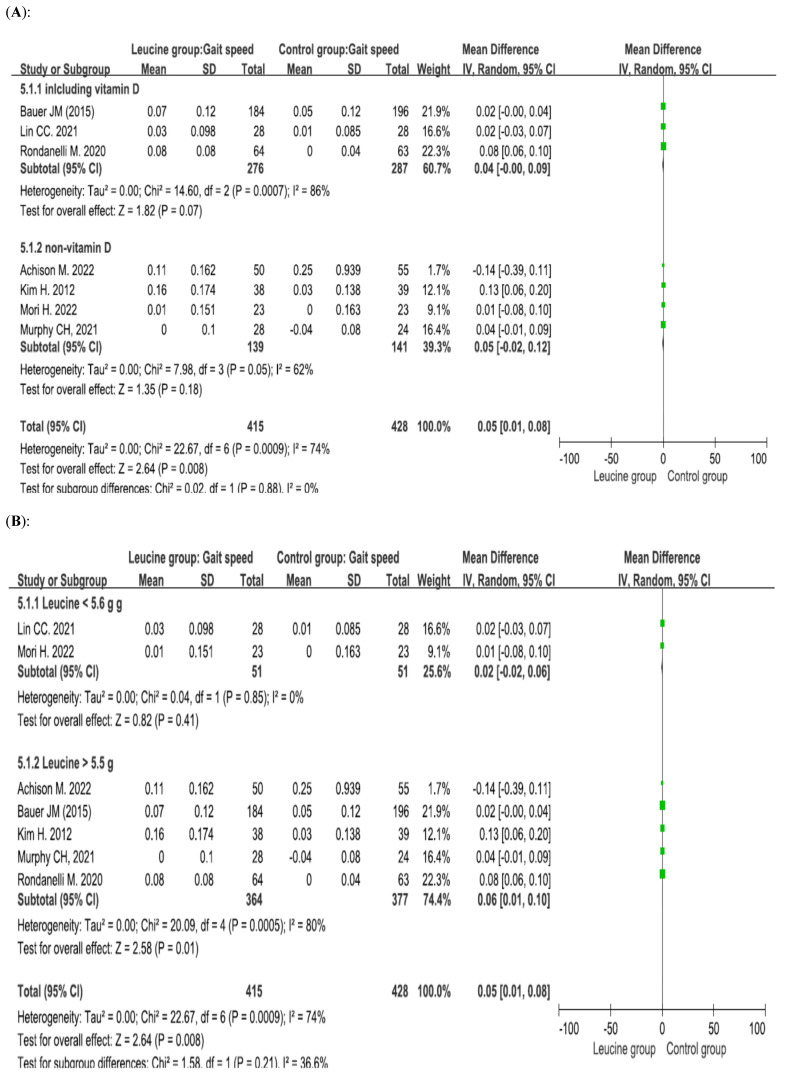
Forest plots assessing the effect of leucine supplementation on gait speed in patients with sarcopenia by (**A**) vitamin D supplementation status and (**B**) leucine dose [32,34,35,45,46,48,50].

Seven studies involving 843 patients (415 patients receiving leucine and 428 control patients) reported gait speed, and the difference between the groups was significant (*p* = 0.008, mean difference = 0.05) [32,34,35,45,46,49,50]. Subgroup analysis of patients who concurrently received vitamin D was performed. When vitamin D was combined with leucine, there was no significant difference between the leucine group and the control group (*p* = 0.07, mean difference = 0.04). When vitamin D was not combined with leucine, there was no significant difference between the leucine group and the control group (*p* = 0.18, mean difference = 0.05) (Figure 6A). Subgroup analysis of the daily amount of leucine was performed. When patients received a low dose of leucine, there was no significant difference between the leucine group and the control group (*p* = 0.41, mean difference = 0.02). When patients received a high dose of leucine, there was a significant difference between the leucine group and the control group (*p* = 0.01, mean difference = 0.06) (Figure 6B).

## 4. Discussion

The present meta-analysis of eleven trials revealed that patients receiving leucine and patients receiving a placebo had significantly different handgrip, ASMM/height^2^, and gait speed values. The SPPB scores of patients receiving leucine and those receiving placebo did not significantly vary.

### 4.1. Effect of Leucine Supplementation on Handgrip Strength

Guo Y et al.’s meta-analysis of a total of 17 RCTs with 1418 older participants revealed that leucine supplementation alone did not increase older people’s handgrip strength [41]. The meta-analysis conducted by Chang MC et al. included three RCTs with a total of 637 participants. The findings indicated that the implementation of a concomitant physical exercise program resulted in a significant improvement in handgrip strength in the experimental group compared with the control group. Conversely, the absence of physical exercise did not result in a significant increase in handgrip strength in patients with sarcopenia [51]. The meta-analysis conducted by Lee SY et al. included six RCTs involving a total of 699 older sarcopenic adults. Compared with that of the control group, the overall muscle strength of the group that received leucine-rich protein was increased [52]. In the present meta-analysis, only two of the nine studies reported that leucine supplementation did not increase handgrip strength in elderly sarcopenic patients [34,50]. In the study by Achison M et al., not all older sarcopenic adults received any combination therapy, including exercise training and vitamin D supplementation [34]. In the study by Lin CC et al., all of the older sarcopenic adults did not receive exercise training and all of the older sarcopenic adults received a low dose of leucine [38]. Seven other studies revealed that leucine supplementation tended to increase handgrip strength in older sarcopenic adults [31,32,35,46,47,48,49]. Statistical significance was not consistently observed across subgroups. We concluded that there is evidence suggesting a trend of older sarcopenic adults receiving leucine supplementation having increased handgrip strength as a result.

### 4.2. Effect of Leucine Supplementation on the ASMM/Height^2^

A meta-analysis by Guo Y et al. revealed that supplementation with leucine alone did not increase muscle mass in older people [41]. A meta-analysis by Chang MC et al. revealed that regardless of whether a concurrent physical activity program was provided, appendicular muscle mass significantly increased [51]. A meta-analysis by Lee SY et al. revealed that supplementation with leucine-rich proteins tended to increase participants’ muscle mass relative to that of the control; however, the differences were not statistically significant [52]. In the present meta-analysis, all five studies reported that leucine supplementation improved the ASMM/height^2^ in elderly sarcopenic patients [35,47,48,49,50]. We concluded that there is evidence suggesting a trend of older sarcopenic adults receiving leucine supplementation having improved ASMM/height^2^ as a result.

### 4.3. Effect of Leucine Supplementation on the SPPB Score

A meta-analysis by Guo Y et al. revealed that supplementation with leucine alone did not improve older people’s SPPB scores [41]. A meta-analysis by Chang MC et al. revealed that, compared with the control group, the experimental group’s SPPB scores significantly improved with the implementation of a concurrent physical training program. On the other hand, the SPPB scores of the sarcopenic patients did not significantly improve when a physical exercise program was not provided [51]. The present meta-analysis revealed no significant differences in SPPB scores between the leucine supplementation group and the control group. The SPPB score may have been influenced by other parameters, such as fatigue and neurological, cardiovascular, and respiratory problems, which could have altered the results of the test [51].

### 4.4. Effect of Leucine Supplementation on Gait Speed

A meta-analysis by Guo Y et al. revealed that supplementation with leucine and vitamin D significantly improved gait speed in older individuals [41]. A meta-analysis by Lee SY et al. revealed that leucine-rich protein supplementation tended to enhance participants’ physical performance (SPPB scores, gait speed, physical fitness test results, etc.) compared with that of the control intervention, but the differences were not statistically significant [52]. In the systematic review by Martínez-Arnau FM et al., three of the six studies that assessed the effect of dietary leucine supplementation on physical performance (as measured by walking speed) reported a significant improvement [29]. In the present meta-analysis, only Achison M et al. reported that leucine supplementation did not increase gait speed in elderly sarcopenic patients [34]. Six other studies revealed that leucine supplementation tended to increase gait speed in older sarcopenic adults [32,35,45,46,48,50]. We concluded that there is evidence suggesting a trend of older sarcopenic adults receiving leucine supplementation having increased gait speed as a result.

### 4.5. Leucine Dosage

The international guidelines recommend that the goal is to counteract the loss of lean muscle mass in elderly individuals through the consumption of 3 g of leucine in three main meals together with 25–30 g of protein [53,54]. A nutritional recommendation is for elderly individuals to consume more protein (1–1.2 g/kg/day). It is recommended that leucine be taken at least twice a day at a maximum dose of 2.8–3 g [55,56]. The present meta-analysis only revealed that when patients received a high dosage of leucine, there was a significant difference in the ASMM/height^2^ and gait speed between the high dosage of the leucine group and the control group.

### 4.6. Vitamin D Supplementation

According to the International Osteoporosis Foundation, elderly people who consume 800–1000 IU of vitamin D daily have better muscular function, stronger muscles, and a lower risk of fractures [57,58]. A meta-analysis by Guo Y et al. revealed that supplementation with leucine and vitamin D significantly increased handgrip strength and gait speed in older adults [41]. Moreover, monotherapy with vitamin D supplementation in sarcopenic adults had no effect on muscle mass, strength, or performance. Leucine combined with vitamin D may be more beneficial than leucine or vitamin D alone [30]. In the present meta-analysis, only the ASMM/height^2^ significantly differed between the leucine group receiving vitamin D and the control group.

### 4.7. Limitations

Our meta-analysis has several limitations. Surprisingly, only a few RCTs met our requirements for inclusion and there was moderate-to-high risk of bias in many included studies. Consequently, the data collected for this study were subpar. The studies we included in our meta-analysis having an extremely small sample size was another disadvantage, as this inherently reduces the statistical power. There was a lack of consistency across the included studies in terms of the participants (older individuals with sarcopenia and healthy older adults), high variability in leucine supplementation dose, concurrent combined treatment regimen, study duration, and outcome definitions. ASMM/height^2^ and gait speed were consistent, but grip strength and SPPB were assessed using different protocols across studies, which was another limitation. The included studies varied in their reported raw values of grip strength. We could not collect the raw values from all included studies, and we could not standardize units across studies or convert them to standardized mean differences for comparability. This was also a limitation. Multiple subgroup comparisons without adjustment were noted, and we could not perform a hierarchical plan or Bonferroni correction, which was a noted limitation. Moreover, the use of a fixed correlation coefficient of 0.8 without sensitivity analysis can overestimate precision. However, we were unable to conduct a sensitivity analysis, which was another limitation. Three Rondanelli trials arose from the same Italian center over short periods, and duplicate enrollment may have occurred. Although it is challenging to draw firm conclusions owing to the variability in the examined research, there is a tendency toward positive effects of leucine supplementation in older sarcopenic patients. More experimental research is needed to elucidate and better comprehend the impact of leucine supplementation.

## 5. Conclusions

Low-quality evidence was found that older sarcopenic patients receiving leucine may show trends toward improved skeletal muscle strength, skeletal muscle quality, and physical performance. The capacity of leucine supplementation to have a beneficial therapeutic impact in older sarcopenic individuals is restricted when it is used alone without concurrent additional therapy. The muscular strength, muscle mass, and physical performance of older sarcopenic individuals tend to be enhanced by combining an exercise training program with high doses of leucine and vitamin D, which needs stronger qualification.

## Figures and Tables

**Table 1:** Characteristics of the included studies.

Author/ Year	Region	Subject Characteristics	Leucine Dose (per Day)	Common Treatment in Both Groups	Duration	Outcomes	No. of Participants (Leucine)	No. of Participants (Control)
Achison M, 2022 [34]	UK	≥70 Y/O	7.5 g	NIL	12 months	Handgrip strength, SPPB, Gait speed	72	72
Amasene M, 2019 [33]	Spain	≥70 Y/O (Post-hospitalized)	3.0 g	Both: RTP	12 weeks	SPPB	15	13
Bauer JM, 2015 [32]	EURO	≥65 Y/O	6.0 g	Leucine: Vitamin D 1600 IU Control: NIL	13 weeks	Handgrip strength, SPPB, Gait speed	184	196
Kim HK, 2012 [45]	Japan	≥75 Y/O (Women)	6.0 g	Both: PMTP	3 months	Gait speed	38	39
Lin CC, 2021 [50]	Taiwan	≥65 Y/O	3.6 g	Leucine: Vitamin D 360 IU Control: NIL	12 weeks	Handgrip strength, ASMM, Gait speed	28	28
Martinez-Arnau F, 2020 [31]	EURO	≥65 Y/O	6 g	NIL	13 weeks	Handgrip strength	23	19
Mori H, 2022 [35]	Japan	≥65 Y/O	2.3 g	Both: RTP	24 weeks	Handgrip strength, ASMM, Gait speed	23	23
Murphy CH, 2021 [46]	EURO	≥65 Y/O	6.2 g	NIL	24 weeks	Handgrip strength, SPPB, Gait speed	28	25
Rondanelli M, 2016 [47]	Italy	≥65 Y/O	4 g	Leucine: vitamin D 100 IU and PMTP. Control: PMTP	12 weeks	Handgrip strength, ASMM,	69	61
Rondanelli M, 2020 [48]	Italy	≥65 Y/O	5.6 g	Leucine: vitamin D 1600 IU Control: NIL	4–8 weeks	Handgrip strength, SPPB, ASMM, Gait speed	64	63
Rondanelli M, 2022 [49]	Italy	≥65 Y/O	2.5 g	Leucine: *Lactobacillus paracasei* PS23; omega-3 fatty acids 500 mg Control: NIL	2 months	Handgrip strength, SPPB, ASMM	22	28

Abbreviations: RTP: resistance training program; ASMM: appendicular skeletal muscle mass; SPPB: short physical performance battery; PMTP: physical fitness and muscle mass enhancement training program; UK: United Kingdom; EURO: European Union; Y/O: years old; NIL: nothing; No.: number.

## Data Availability

The datasets generated and/or analyzed during the current study are not publicly available but are available from the corresponding author on reasonable request.
